# Comparison of intraoperative blood loss in piezoelectric vs. conventional technique surgeries: a systematic review and meta-analysis

**DOI:** 10.3389/froh.2025.1687571

**Published:** 2025-11-27

**Authors:** Sina Ahmadi, Sahar Rajaei, Farnoosh Alimohammadi, Parastoo Ghaderi, Melika Mokhtari, Junbo Tu, Sijia Na

**Affiliations:** 1 Key Laboratory of Shaanxi Province for Craniofacial Precision Medicine Research, College of Stomatology, Xi'an Jiaotong University, Xi'an, Shaanxi, China; 2 Department of Oral and Maxillofacial Surgery, College of Stomatology, Xi'an Jiaotong University, Xi'an, Shaanxi, China; 3 Faculty of Dentistry, Yazd University of Medical Sciences, Yazd, Iran; 4 Department of Oral Medicine, School of Dentistry, Arak University of Medical Sciences, Arak, Iran; 5 Faculty of Dentistry, Tabriz University of Medical Sciences, Tabriz, Iran; 6 Faculty of Medicine, Tehran Medical Sciences, Islamic Azad University, Tehran, Iran

**Keywords:** piezosurgery, orthognathic surgery, blood loss, surgical, surgery, oral, ultrasonic surgical procedures, ultrasonic devices

## Abstract

**Background and aim:**

Orthognathic surgeries often involve significant blood loss due to the high vascularity of the maxillofacial region. Conventional tools such as saws and burrs, while effective, can cause tissue damage and heat that may delay healing. Piezosurgery, a newer technique using ultrasonic vibrations, allows for precise bone cutting while protecting the soft tissues and nerves. This review aimed to compare intraoperative blood loss between piezosurgery and conventional osteotomy methods.

**Materials and methods:**

A systematic search of PubMed, Scopus, and Web of Science was conducted up to 5 October 2025, following the Preferred Reporting Items for Systematic reviews and Meta-Analyses guidelines. Studies involving patients who underwent orthognathic surgery using either piezosurgery or conventional tools, with reported blood loss data, were included. Six studies with 144 participants and 252 surgeries met the criteria. The data were analyzed using STATA 18 to calculate the weighted mean difference (WMD). Heterogeneity was assessed, and publication bias was evaluated with funnel plots.

**Results:**

Piezosurgery significantly reduced blood loss compared to conventional methods, with a WMD of −81.73 mL (95% CI: −97.30 to −66.16, *P* < 0.001). The sensitivity analysis confirmed the reliability of these results, and formal statistical tests indicated no significant publication bias.

**Conclusion:**

Piezosurgery significantly reduces intraoperative blood loss during orthognathic surgeries, likely due to its precision and minimal tissue damage. Although it is more costly and takes longer, it offers a safer alternative, particularly in complex cases. Further research with larger samples is needed to confirm these findings.

**Systematic Review Registration:**

PROSPERO CRD420251232995.

## Introduction

In the context of orthognathic surgeries, osteotomies are performed in close proximity to delicate anatomical structures. Traditionally, saws, burs, and chisels have been utilized to cut bones. While these instruments are highly effective, they can potentially cause damage to the adjacent soft tissues and nerves. Furthermore, the use of these rotating instruments can be injurious, as the production of excessively high temperatures can impair bone regeneration and result in bone necrosis (1–4).

The need for less invasive surgical approaches and greater precision compared to standard bur and saw techniques has led to the development of piezosurgery. This technology was invented by Tomaso Vercellotti and operates on the principle of the “piezoelectric effect.” Presently, piezoelectric devices are utilized extensively for various osteotomies in the field of oral and maxillofacial surgery, including maxillary sinus lift procedures, extraction of impacted mandibular third molars, and bone grafting. This piezosurgical device employs ultrasonic vibrations to selectively remove bone with minimal damage to the surrounding soft tissues, including blood vessels and nerves. In addition, piezosurgery provides excellent visibility due to its cavitation effect (1–3).

Orthognathic surgery often involves significant blood loss due to the rich vascularization of the maxillofacial region (5). Managing bleeding during these procedures can be particularly challenging, as major vessels such as the sphenopalatine artery, descending palatine artery, pterygoid plexus, and internal maxillary artery are frequently at risk (6). Procedures such as Le Fort I osteotomies and bimaxillary surgeries further heighten this risk because of their complexity and duration (7). The intricate anatomy and high vascularity of the area make it difficult to achieve effective hemostasis through traditional methods such as cauterization or vessel ligation (5). As the length and complexity of the surgery increase, so does the likelihood of significant blood loss (8).

This systematic review analyzed studies to compare intraoperative blood loss between piezosurgery and conventional osteotomy techniques in orthognathic procedures.

## Methods

### Search strategy

Two independent authors conducted extensive searches across electronic databases, including PubMed, Scopus, and Web of Science, until 5 October 2025, in accordance with the Preferred Reporting Items for Systematic reviews and Meta-Analyses (PRISMA) guidelines. The search strategy involved a combination of Medical Subject Headings (MeSH) terms and text-based keywords. In addition, the researchers manually reviewed the reference lists of the selected articles and related review papers and meta-analyses to identify any additional potentially relevant publications. The search terms included “ultrasonic surgical procedures” OR “ultrasonic surgery” OR “ultrasonic therapy” OR “ultrasonic cutting” OR “ultrasonic bone cutting” OR “piezosurgery” OR “piezo-electric surgery” OR “piezo-electric bone surgery” OR “piezoelectric osteotomy” OR “ultrasonic surgery procedure” OR “orthognathic surgery” OR “orthognathic surgical procedures” OR “jaw surgery” OR “osteotomy”(. The search strategy for each database is shown in Table 1. This systematic review was registered in the International Prospective Register of Systematic Reviews (PROSPERO) under the registration code CRD420251232995.

**Table 1:** Search strategy.

Database	Query
PubMed	((“ultrasonic surgical procedures”[Title/Abstract] OR “ultrasonic surgery”[Title/Abstract] OR “ultrasonic therapy”[Title/Abstract] OR “ultrasonic cutting”[Title/Abstract] OR “ultrasonic bone cutting”[Title/Abstract] OR “piezosurgery”[Title/Abstract] OR “piezo-electric surgery”[Title/Abstract] OR “piezo-electric bone surgery”[Title/Abstract] OR “piezoelectric osteotomy”[Title/Abstract] OR “ultrasonic surgery procedure”[Title/Abstract]) AND (“orthognathic surgery”[Title/Abstract] OR “orthognathic surgical procedures”[Title/Abstract] OR “jaw surgery”(Title/Abstract) OR “osteotomy”[Title/Abstract]))
Scopus	(TITLE-ABS (“ultrasonic surgical procedures” OR “ultrasonic surgery” OR “ultrasonic therapy” OR “ultrasonic cutting” OR “ultrasonic bone cutting” OR “piezosurgery” OR “piezo-electric surgery” OR “piezo-electric bone surgery” OR “piezoelectric osteotomy” OR “ultrasonic surgery procedure”) AND TITLE-ABS(“orthognathic surgery” OR “orthognathic surgical procedures” OR “jaw surgery” OR “osteotomy”))
Web of Science	(TS = (“ultrasonic surgical procedures” OR “ultrasonic surgery” OR “ultrasonic therapy” OR “ultrasonic cutting” OR “ultrasonic bone cutting” OR “piezosurgery” OR “piezo-electric surgery” OR “piezo-electric bone surgery” OR “piezoelectric osteotomy” OR “ultrasonic surgery procedure”) AND TS = (“orthognathic surgery” OR “orthognathic surgical procedures” OR “jaw surgery” OR “osteotomy”))

### Inclusion criteria and study selection

To be included in the meta-analysis, the studies had to meet the following pre-established criteria:

1. Written in English;
2. Population of the study: patients who underwent orthognathic surgery;
3. Including at least one of the following two groups: piezoelectric osteotomy or conventional osteotomy;
4. Examining intraoperative blood loss; and
5. Study type: observational studies and controlled trials.

The PICOT (Patient, Intervention, Comparison, Outcome, Time) elements were considered to determine the eligibility of the studies.

Following the removal of duplicate publications, two researchers (A and B) independently screened the remaining articles based on their titles and abstracts to assess their eligibility for inclusion, using a comprehensive search strategy with broad criteria. After the initial screening, they reviewed the full-text articles, and their eligibility was decided through a consensus process. Any disagreements were resolved through discussion among the reviewers.

### Data extraction

Two researchers (A and B) collaborated to ensure the accuracy of the data extraction process. They extracted data using a predesigned data collection spreadsheet (Microsoft Excel). The following information was extracted from each article for comparative purposes: author, country of origin, journal, year of publication, study population, age, sample size, type of surgical procedure, name of intervention, name of comparison group, and outcomes.

### Risk of bias assessment

The risk of bias in the included studies was assessed using the guidelines in the JBI (Joanna Briggs Institute) critical appraisal tool. Two independent researchers (A and B) conducted the quality assessment of all the studies in the review. Any disagreements between their evaluations were discussed to reach an agreement. In instances where researchers A and B could not resolve the discrepancy, a third researcher, C, was consulted to intervene and help determine the final quality rating for the disputed study.

### Statistical analysis

A meta-analysis was conducted using STATA version 18 software (StataCorp, College Station, TX) to compare intraoperative blood loss in orthognathic surgery performed using piezosurgery vs. conventional osteotomy techniques. The weighted mean difference (WMD) with a 95% confidence interval (95% CI) was used to pool the individual study results for the blood loss amount. The WMD was calculated using the generic inverse variance method, extracting the means and standard deviations (SDs) for each study group and outcome of interest. The heterogeneity among the included studies was evaluated using the chi-square and I-square tests. Furthermore, to investigate the sources of heterogeneity, subgroup analyses were performed based on the type of surgical procedure [bilateral sagittal split osteotomy (BSSO), Le Fort I osteotomy, and their combination] and geographic location (continent). The differences between the subgroup means were evaluated using the Qb test (test of group differences). All the statistical analyses were conducted using a two-tailed approach, with statistical significance set at a *p*-value less than 0.05.

### Publication bias assessment

The researchers evaluated the meta-analysis for the potential presence of publication bias using a visual inspection of the funnel plot and conducted formal statistical tests, specifically Egger's regression test and Begg's correlation test.

### Sensitivity analysis

The researchers also conducted a sensitivity analysis as part of the meta-analysis. The sensitivity analysis evaluated whether the results were heavily dependent on any single study included in the review. This involved the leave-one-out method, where each study was sequentially removed, and the impact on the overall findings and conclusions of the meta-analysis was assessed (9).

## Results

**Figure 1:**
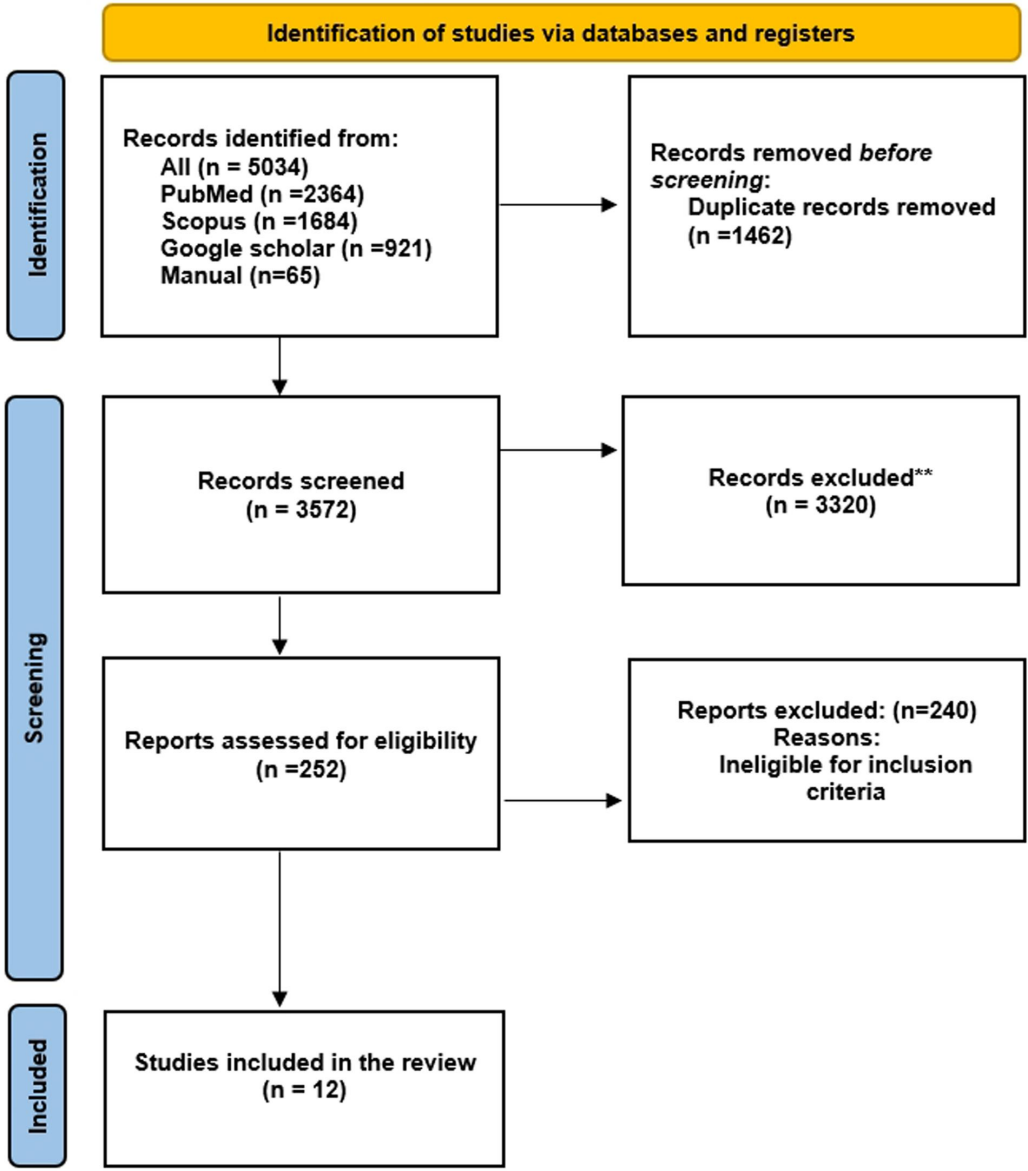
PRISMA flow diagram for this systematic review.

A comprehensive search initially identified 1,120 studies. After removing 842 duplicates, the abstracts of 278 studies were screened. During this process, 263 studies were excluded due to not meeting the inclusion criteria. The full texts of 19 studies were then reviewed, with nine excluded for the reasons detailed in Figure 1. Ultimately, six studies were deemed eligible and included in the meta-analysis. The six included studies encompassed 144 participants and reported on 252 orthognathic surgeries, including BSSOs, Le Fort I osteotomies, and genioplasties. These studies, published between 2014 and 2025, were conducted in Italy, Pakistan, Turkey, India, Japan, and Egypt. Each study compared piezosurgery with conventional cutting techniques, specifically evaluating intraoperative blood loss in orthognathic procedures. The key characteristics of these studies are summarized in Table 2.

**Table 2:** Characteristics of included studies.

Study	Country	Sample size	Age (mean ± SD)	Gender (female)	Blood loss (mL)	Type of surgery	Surgeries (*n*)
Sato et al., 2023 (29)	Japan	Piezosurgery: 10	Piezosurgery: 19.2	Piezosurgery: 3	230 ± 45.15 (piezosurgery)	BSSO and Le Fort I	20
		Conventional: 10	Conventional: 19.1	Conventional: 5	343 ± 49.463 (conventional)		
Barakat et al., 2025 (30)	Egypt	Piezosurgery: 5	22.5	10 (100%)	38.30 ± 4.50 (piezosurgery)	BSSO	10
		Conventional: 5			114.70 ± 14.35 (conventional)		
Spinelli et al., 2014 (15)	Italy	12	26.08 ± 5.56	7 (58%)	237.50 ± 87.08 (piezosurgery)	BSSO and Le Fort I	12
					311.67 ± 97.03 (conventional)		12
Akbar et al., 2015 (16)	Pakistan	24	21.96 ± 1.42	12 (50%)	212 ± 32.5 (piezosurgery)	Le Fort I Osteotomy, BSSO	48
					315 ± 52.5 (conventional)		48
Demirbas et al., 2015 (17)	Turkey	Piezosurgery: 12	Piezosurgery: 22.1 ± 3.3	Piezosurgery: 10 (83%)	157.5 ± 61.51 (piezosurgery)	Le Fort I osteotomy	12
		Conventional: 12	Conventional: 20.9 ± 2.4	Conventional: 6 (50%)	194.16 ± 107.14 (conventional)		12
Pattabhi et al., 2024 (18)	India	Piezosurgery: 30	NR	NR	110 ± 25 (piezosurgery)	BSSO	30
		Conventional: 30			180 ± 30 (conventional)		30

NR, not reported.

### Quality assessment

The quality of the included studies was evaluated using the JBI Critical Appraisal Tools, with the results summarized in Table 3.

**Table 3:** Risk of bias: critical appraisal tool for cohort studies.

Checklist question	Demirbas et al. (17)	Akbar et al. (16)	Spinelli et al. (15)	Pattabhi et al. (18)	Sato et al. (29)	Barakat et al. (30)
1. Were the two groups similar and recruited from the same population?	Yes	Yes	Yes	Yes	Yes	Yes
2. Were the exposures measured similarly to assign people to both exposed and unexposed groups?	Yes	Yes	Yes	Yes	Yes	Yes
3. Was the exposure measured in a valid and reliable way?	Unclear	Yes	Yes	Yes	Yes	Yes
4. Were confounding factors identified?	Yes	Yes	Yes	Yes	Yes	Yes
5. Were strategies to deal with confounding factors stated?	Yes	Unclear	Yes	Unclear	Unclear	Yes
6. Were the groups/participants free of the outcome at the start of the study?	N/A	Yes	Yes	Yes	Yes	Yes
7. Were the outcomes measured in a valid and reliable way?	Yes	Yes	Yes	Yes	Yes	Yes
8. Was follow-up time reported and long enough for outcomes to occur?	Yes	Yes	Yes	Yes	Yes	Yes
9. Was follow-up complete, and if not, were the reasons for loss to follow-up described and explored?	Yes	Yes	Yes	Yes	Yes	Yes
10. Were strategies to address incomplete follow-up utilized?	No	Unclear	No	No	No	No
11. Was appropriate statistical analysis used?	Yes	Yes	Yes	Yes	Yes	Yes

### Blood loss comparison

This systematic review analyzed blood loss during orthognathic surgeries performed with piezoelectric surgical techniques vs. conventional osteotomy instruments. Blood loss was measured consistently across all studies as the total fluid collected in milliliters using a calibrated suction device, subtracting the volume of saline irrigation to ensure accuracy. The results are reported as means with standard deviations and 95% CIs.

**Figure 2:**
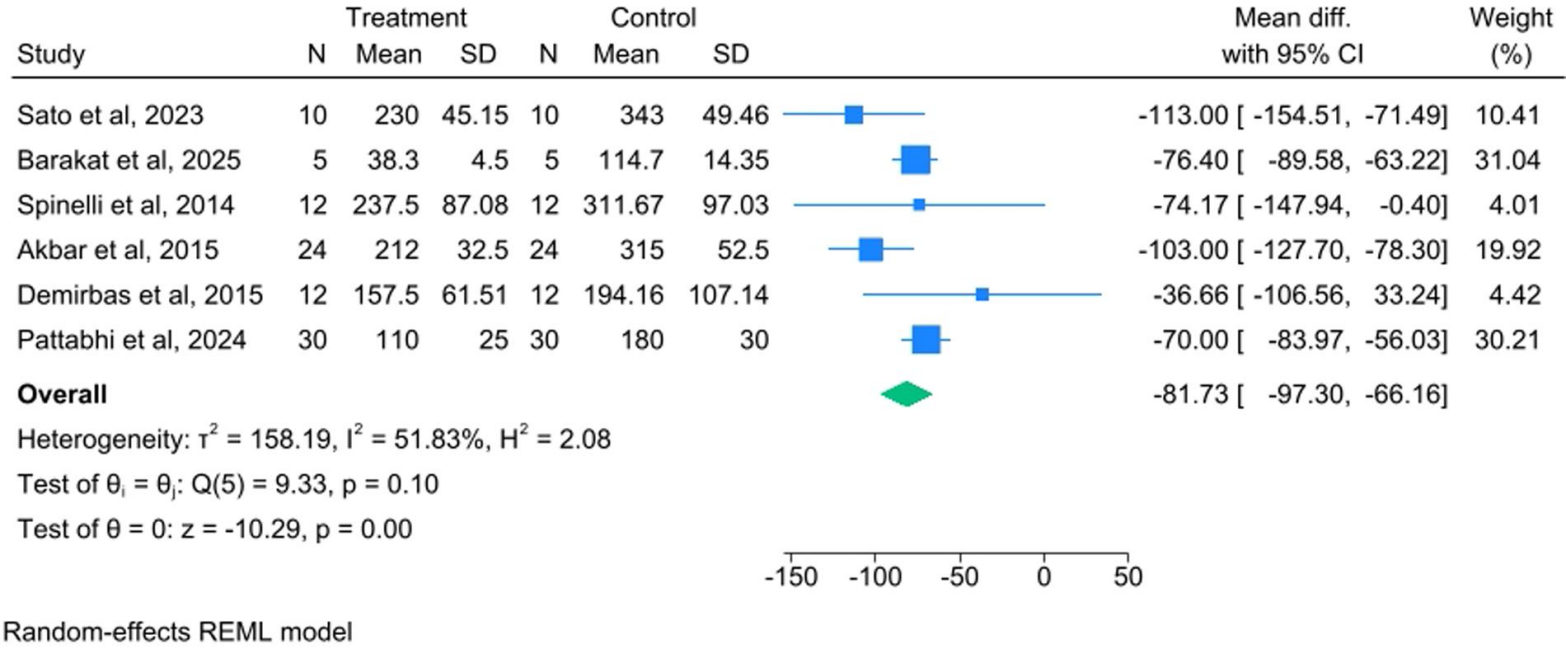
Forest plot comparing the mean difference in intraoperative blood loss between piezosurgery and conventional cutting techniques (random effects model).

The meta-analysis demonstrated that piezosurgery significantly reduced intraoperative blood loss compared to conventional cutting methods. The overall WMD was −81.73 mL (95% CI: −97.30 to −66.16, *P* < 0.001), as shown in Figure 2. The observed heterogeneity was *I*^2^ = 51.83% [Test of *θi* = *θj*:Q(5) = 9.33, *p* = 0.10].

### Subgroup analysis

#### Subgroup analysis by procedure type

**Figure 3:**
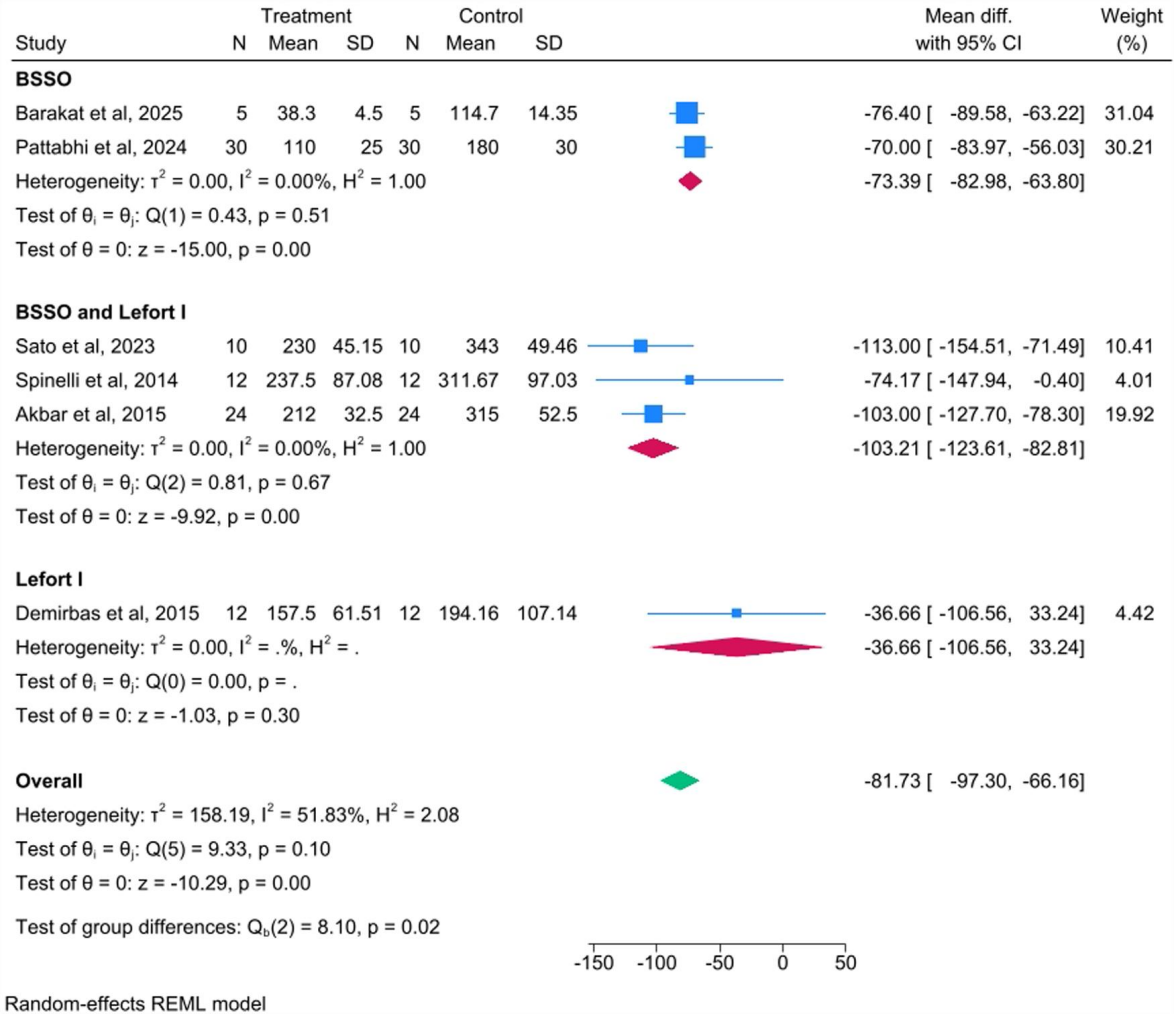
Subgroup analysis by type of surgery.

A subgroup analysis was performed based on the type of orthognathic procedure performed (Figure 3). The most substantial reduction in blood loss was observed in studies including the BSSO and Le Fort I procedures, with a WMD of −103.21 mL (95% CI: −123.61 to −82.81). In the studies that focused only on BSSOs, the WMD was −73.39 mL (95% CI: −82.98 to −63.80). The single study that focused only on Le Fort I osteotomies showed a WMD of −36.66 mL (95% CI: −106.56 to 33.24). The test for differences between the subgroup means was statistically significant [Qb (2) = 8.10, *p* = 0.02]. Importantly, heterogeneity was completely eliminated in all the procedural subgroups (*I*^2^ = 0.00% for all three subgroups).

#### Subgroup analysis by geographic location

**Figure 4:**
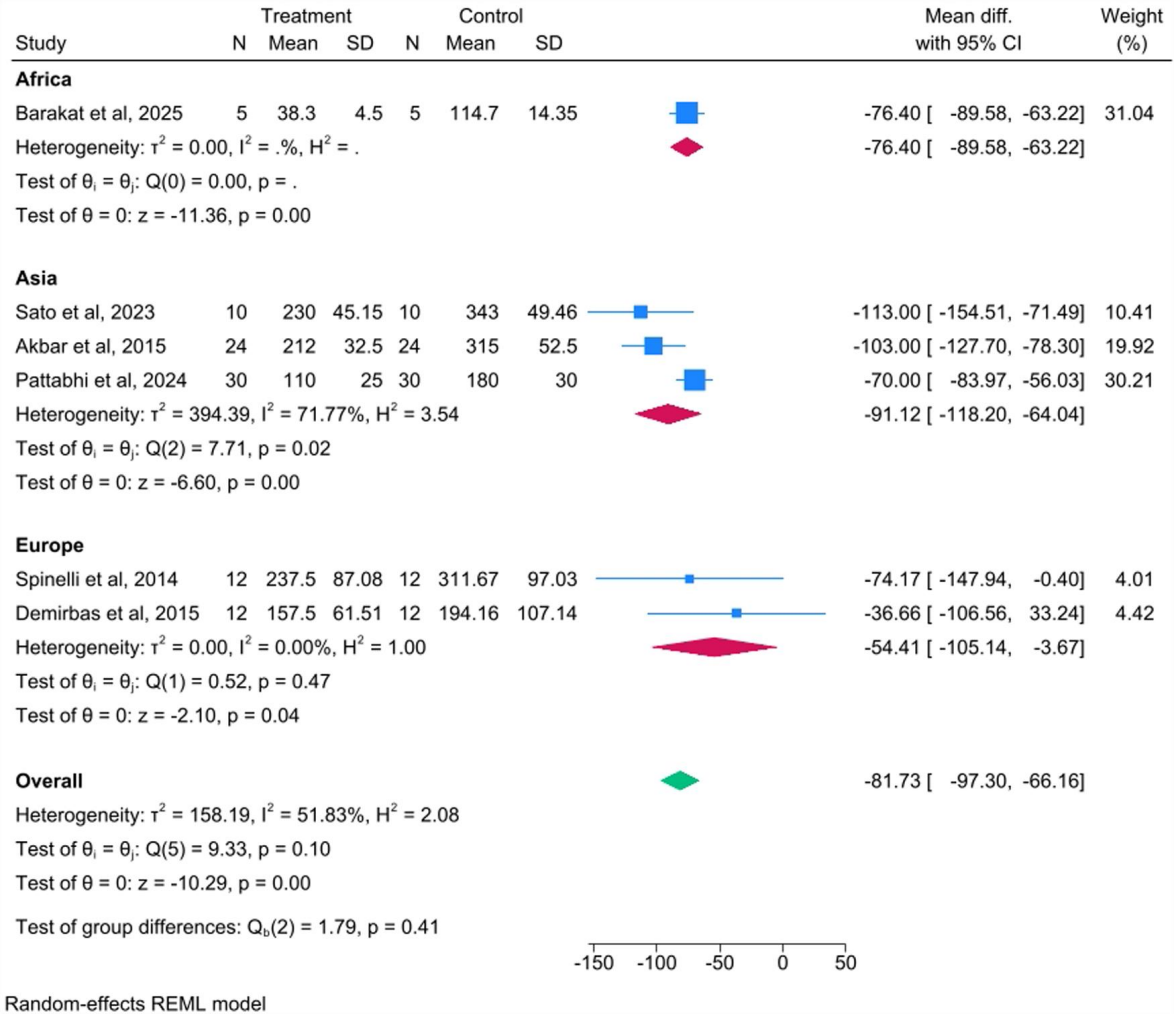
Subgroup analysis by geographic location.

A second subgroup analysis was performed based on the geographic region where the study was conducted (Figure 4). The largest reduction in blood loss was observed in the studies from Asia, with a WMD of −91.12 mL (95% CI: −118.20 to −64.04). The studies from Europe showed a WMD of −54.41 mL (95% CI: −105.14 to −3.67). The single study from Africa showed a WMD of −76.40 mL (95% CI: −89.58 to −63.22). The test for differences between the subgroup means was not statistically significant [Qb (2) = 1.79, *p* = 0.41].

### Sensitivity and bias analysis

**Figure 5:**
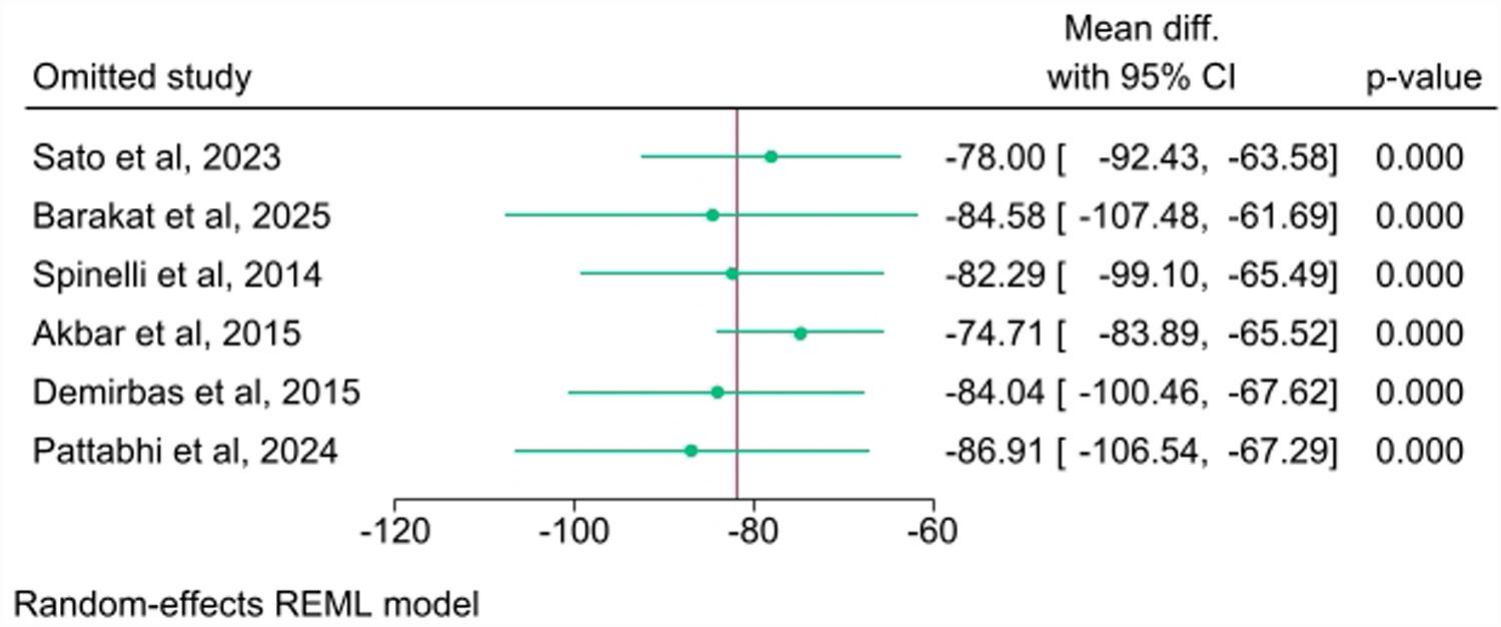
Leave-one-out analysis plot for the included studies.

A sensitivity analysis was conducted to determine whether individual studies had an undue influence on the pooled results. The sensitivity analysis (Figure 5) was performed using the leave-one-out method and demonstrated consistent pooled estimates when each study was sequentially omitted. The overall WMD remained statistically significant (*p* < 0.001 for all iterations) and varied only slightly, ranging from a minimum of −74.71 mL [when Akbar et al. (2015) was omitted] to a maximum of −86.91 mL [when Pattabhi et al. (2024) was omitted]. This confirms the robustness and reliability of the meta-analysis findings.

**Figure 6:**
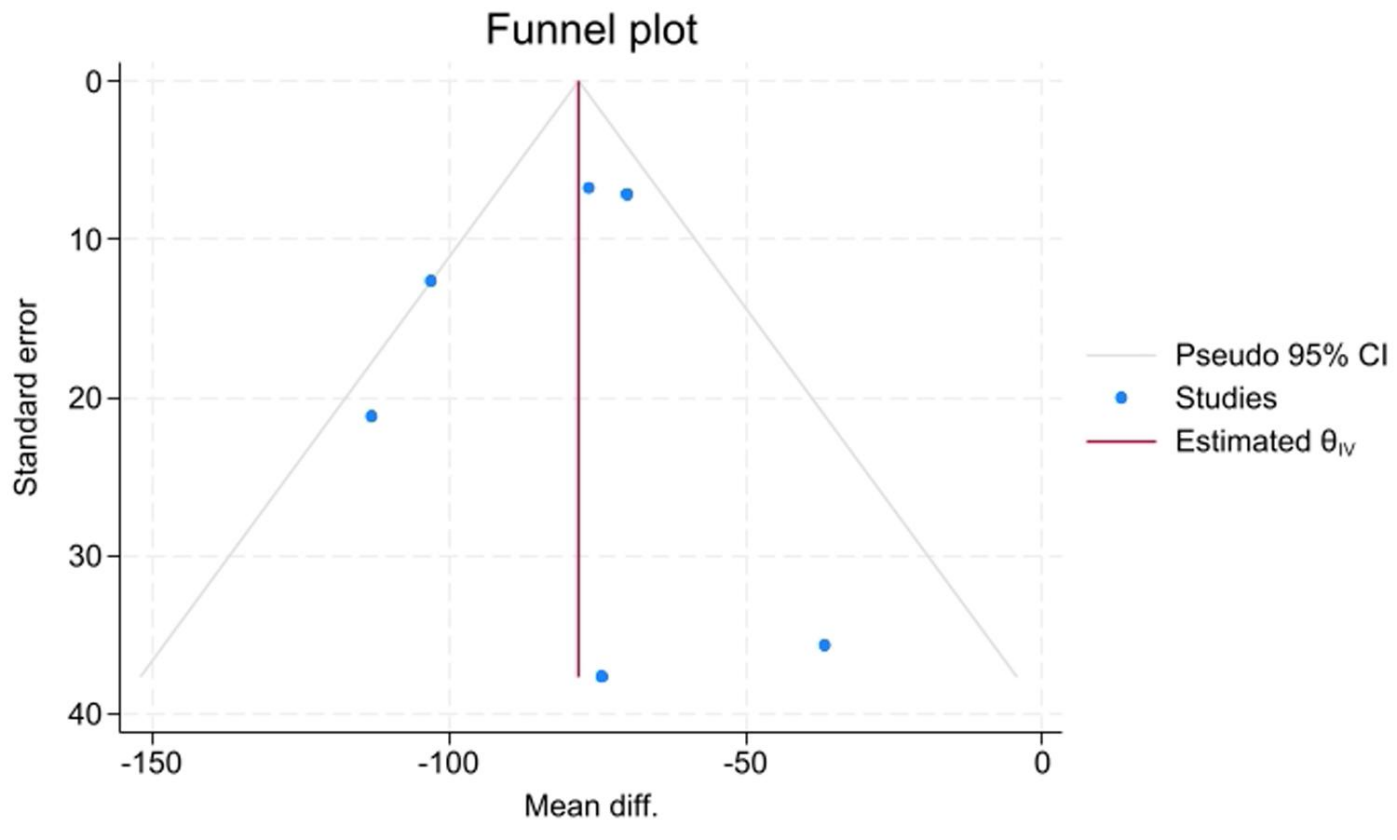
Funnel plot for the comparison of intraoperative blood loss between piezosurgery and conventional cutting.

Publication bias was assessed using a visual inspection of the funnel plot (Figure 6) and formal statistical tests. Visually, the funnel plot showed a degree of asymmetry. However, the formal statistical tests, namely, Egger's regression test (*P* = 0.9385) and Begg's correlation test (*P* = 1.0000), did not show statistically significant evidence of publication bias (small-study effects).

## Discussion

This systematic review compares intraoperative blood loss in piezoelectric vs. conventional technique surgeries. The results consistently demonstrate that piezosurgery offers a significant reduction in blood loss compared to traditional methods. The growing preference for piezosurgery in the field of orthognathic surgery is largely attributed to its minimally invasive nature, selective interaction with tissues, and enhanced safety profile, despite certain limitations that should be considered.

A key advantage of piezoelectric devices is their precision in cutting. Operating at ultrasonic frequencies (24–29 kHz), these devices target mineralized tissues, while sparing the surrounding soft tissues and blood vessels (10, 11). This selective cutting ability minimizes accidental damage to the surrounding structures, reduces bleeding, and provides better visibility in the surgical field, all of which are crucial during complex procedures such as orthognathic surgery (12–14). All the studies included in this review reported lower intraoperative blood loss with piezosurgery when compared to conventional techniques.

While the WMD of −81.73 mL (approximately 82 mL) was statistically significant, its clinical importance must be carefully considered. The moderate overall heterogeneity (*I*^2^ = 51.83%) suggests variability in the treatment effect across studies. This variability was successfully explored through the subgroup analysis by procedure type, which revealed a significant difference in the WMD between the subgroups (*p* = 0.02) and eliminated heterogeneity within each procedural subgroup (*I*^2^ = 0.00%). This finding suggests that the type of orthognathic surgery was a significant source of the initial heterogeneity and confirms that the blood loss reduction with piezosurgery was most pronounced in combined BSSO and Le Fort I procedures (WMD: −103.21 mL). Conversely, the subgroup analysis by geographic location was not statistically significant (*p* = 0.41), suggesting that regional practice differences or patient populations do not significantly influence the outcome.

In contrast, conventional surgical methods have significant drawbacks, especially in terms of heat generation. The use of traditional saws and burrs generates substantial heat—up to 200°C—which can cause thermal damage to nearby cells and tissues (2). Furthermore, these conventional methods offer limited visibility during surgery and increase the risk of soft tissue injury (2).

Several studies have directly compared the outcomes of piezosurgery and conventional techniques. Spinelli et al. (15) conducted a study among 12 patients who underwent bimaxillary procedures (BSSO and Le Fort I). They found a significant 25% reduction in intraoperative blood loss with piezo-osteotomy when compared to the conventional saw technique. Similarly, Akbar et al. (16) found that piezosurgery led to significantly reduced intraoperative blood loss and lower rates of nerve damage.

Although piezosurgery required a longer operative time (63 min) compared to conventional saws, it was considered a safer, less invasive alternative for minimizing blood loss and reducing postoperative complications in bimaxillary surgeries. A more recent prospective study found that piezosurgery reduced bleeding compared to conventional techniques, though the difference did not reach statistical significance (17). In another retrospective study among patients who underwent BSSOs, piezosurgery showed a 39% reduction in intraoperative blood loss compared to traditional cutting techniques, with the difference being statistically significant (18).

Aside from the reduction in intraoperative bleeding, the minimally invasive properties of piezosurgery also contribute to decreased postoperative bleeding, edema, and nerve damage (15–18). These advantages are particularly beneficial for procedures such as Le Fort I osteotomies and bimaxillary surgeries, where major vessels, including the sphenopalatine and descending palatine arteries, are at high risk (5). In contrast, hemorrhage rates in conventional methods have been documented to range from 1.2% to 3% (19–21).

Thus, blood loss during orthognathic surgery can be significant, as the maxillofacial region is richly supplied with blood vessels. The challenges of controlling bleeding during surgery arise from the highly vascular nature of this area and the difficulty in using cauterization or vessel ligation effectively (6). The bleeding typically arises from major vessels, such as the sphenopalatine artery, the descending palatine artery, the pterygoid plexus, and the internal maxillary artery, which are crucial during procedures such as Le Fort I osteotomies (7). The risk of significant bleeding increases with bimaxillary surgeries, where both the complexity and duration of the procedure directly influence the amount of blood loss (22).

Despite its many advantages, piezosurgery has some limitations. The main drawback is the longer cutting time, as piezoelectric devices usually have less cutting power than traditional saws and burrs (23, 24). This slower process can extend the surgery and make lengthy procedures more tiring for surgeons. In addition, the high cost of piezoelectric equipment and the need for specialized training to operate it can make adoption difficult, especially in settings with limited resources (25–27). Furthermore, piezosurgery is not suitable for patients with pacemakers, which further limits its use in certain populations (25). Moreover, the absence of a meta-analysis on operative time is another significant limitation, due to the inconsistent and inadequate reporting in the primary studies. Finally, this review was limited by inconsistent reporting of potential confounders and by focusing solely on electronic databases (excluding gray literature and trial registries), which may affect the completeness of the evidence. More research with larger samples and longer follow-up periods is necessary to verify these findings and examine the long-term benefits of piezosurgery.

However, the findings of this review suggest that piezosurgery is particularly advantageous in complex orthognathic surgeries, especially those involving high vascularity, such as bimaxillary operations. By significantly reducing intraoperative blood loss, piezosurgery minimizes the need for transfusions and the associated risks (18). In addition, the reduced rate of neurosensory disturbances reported with piezosurgery may lead to improved postoperative recovery and higher patient satisfaction levels (28). These advantages make piezosurgery an appealing option for many patients undergoing complex facial surgeries.

In conclusion, piezosurgery significantly reduces intraoperative blood loss in orthognathic surgeries compared to traditional osteotomy methods, likely due to its precision and minimal soft tissue trauma. This suggests it is a promising and potentially safer option for high-risk procedures. The subgroup analysis by procedure type explained most of the methodological differences and showed that blood loss reduction was most notable in combined BSSO and Le Fort I surgeries. The absence of publication bias strengthens these findings, but more research is needed to better understand the clinical importance of blood loss reduction and to accurately assess its cost-effectiveness. Future studies with larger, more consistent samples are necessary to confirm these results, better evaluate clinical relevance, and thoroughly analyze cost-efficiency by including operative time and equipment costs. Although the lack of publication bias supports the findings, more studies remain essential.

## Data Availability

The original contributions presented in the study are included in the article/Supplementary Material, further inquiries can be directed to the corresponding authors.
